# Supplementary material for: Booster Vaccination Against Invasive Pneumococcal Disease and Hepatitis B in Previously Vaccinated Solid Organ Transplant Recipients Without Seroprotection
Source: Vaccines (Basel). 2025 Dec 17;13(12):1253. doi: 10.3390/vaccines13121253 (PMC12737362; doi:10.3390/vaccines13121253)
Supplement: Supplementary file 1 [file vaccines-13-01253-s001.zip › vaccines-4022200-supplementary.pdf]

Supplementary Materials.

Table S1. Median pneumococcal IgG concentrations before and after booster vaccination for 12 examined serotypes. IQR, interquartile range; Tx, transplantation.

Serotype	Post-Tx serology (µg/mL) Median (IQR)	Post-booster serology (µg/mL) Median (IQR)	*p*
**1**	0.19 (0.12-0.46)	0.52 (0.24-1.76)	<0.001
**3**	0.13 (0.07-0.20)	0.25 (0.14-0.69)	<0.001
**4**	0.39 (0.19-0.71)	0.72 (0.28-1.45)	<0.001
**5**	0.18 (0.10-0.44)	0 66 (0.29-1.07)	<0.001
**6B**	0.52 (0.26-0.96)	1.13 (0.42-1.98)	<0.001
**7F**	0.28 (0.10-0.68)	0.76 (0.41-1.71)	<0.001
**9V**	0.42 (0.25-0.68)	0.79 (0.29-1.39)	<0.001
**14**	1.87 (1.15-4.33)	3.96 (1.79-10.59)	<0.001
**18C**	1.21 (0.63-1.72)	1.29 (0.62-3.77)	0.002
**19A**	0.49 (0.22-0.79)	1.42 (0.77-2.84)	<0.001
**19F**	1.86 (1.37-3.07)	3.63 (2.51-5.67)	<0.001
**23F**	0.74 (0.28-1.22)	1.74 (0.62-2.95)	<0.001

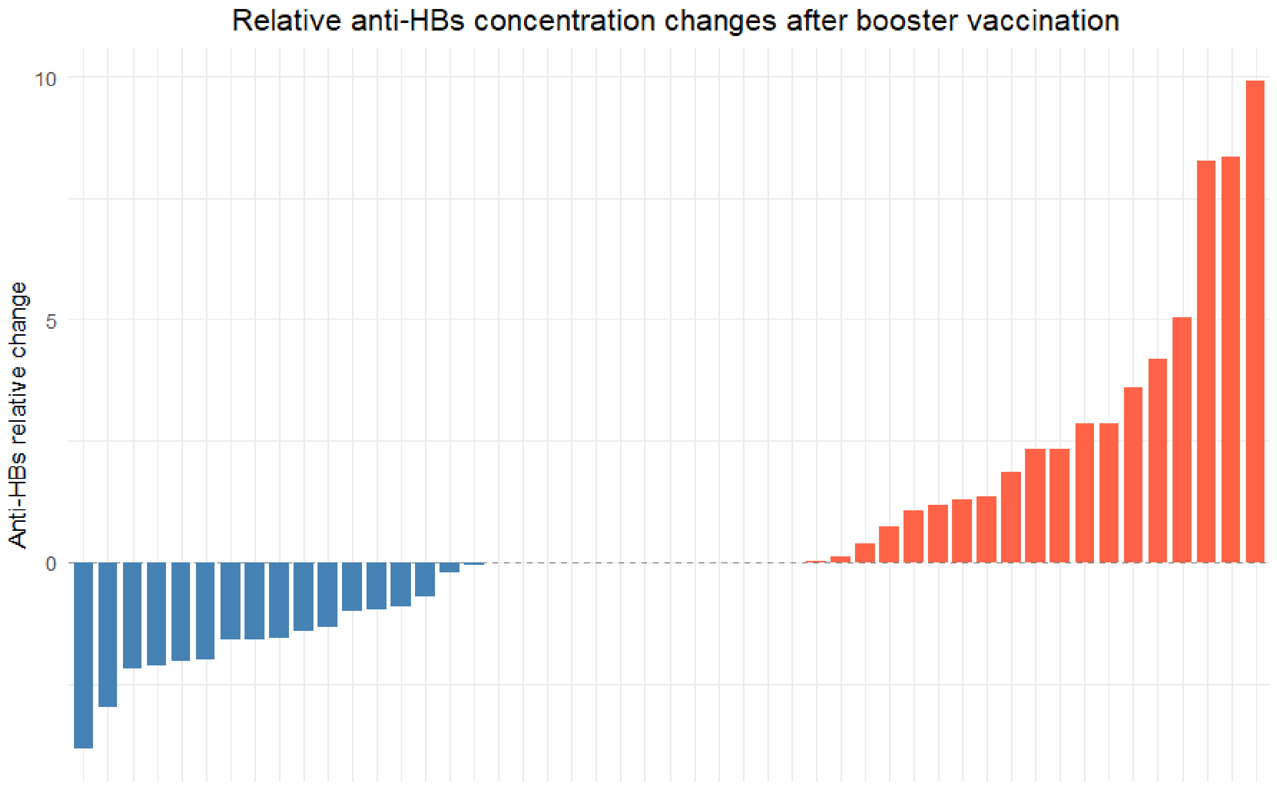

Figure S1. Relative anti-HBs concentration changes after booster vaccination. Plot showing the log_2 transformed relative changes in anti-HBs concentrations after the first booster vaccine compared to before booster vaccine. Each bar on the x-axis represents one SOT recipient. The y-axis represents log_2 transformed

relative changes. Positive changes are shown in red, negative changes are shown in blue.

Table S2. Risk factors associated relative anti-HBs concentration changes after booster vaccination. β represents the estimated fold-changes after hepatitis B booster vaccination compared to before booster vaccination. CI, confidence interval.

	**Unadjusted model**			**Adjusted model***		
	**β**	**95% CI**	***p***	**aβ**	**95% CI**	***p***
**Age (per year of age)**	0.97	0.99; 1.07	0.125			
**Vaccine series completed post-transplantation**	0.43	0.14; 1.33	0.140			
**Seroprotection at time of pre-transplantation vaccination**	1.67	0.03; 84.6	0.794	3.65	0.07; 196	0.516
**Male sex**	0.48	0.16;1.41	0.176	0.37	0.15; 1.29	0.131
**Time between transplantation and booster (per year)**	1.40	0.63; 3.12	0.403	1.84	0.82; 4.12	0.136
**Time between booster and blood sample (per month)**	0.98	0.92; 1.04	0.458	0.99	0.93; 1.06	0.797
**Diabetes**	2.00	0.37; 10.7	0.413	1.36	0.26; 7.03	0.709

***Adjusted for age and vaccination series completion status**

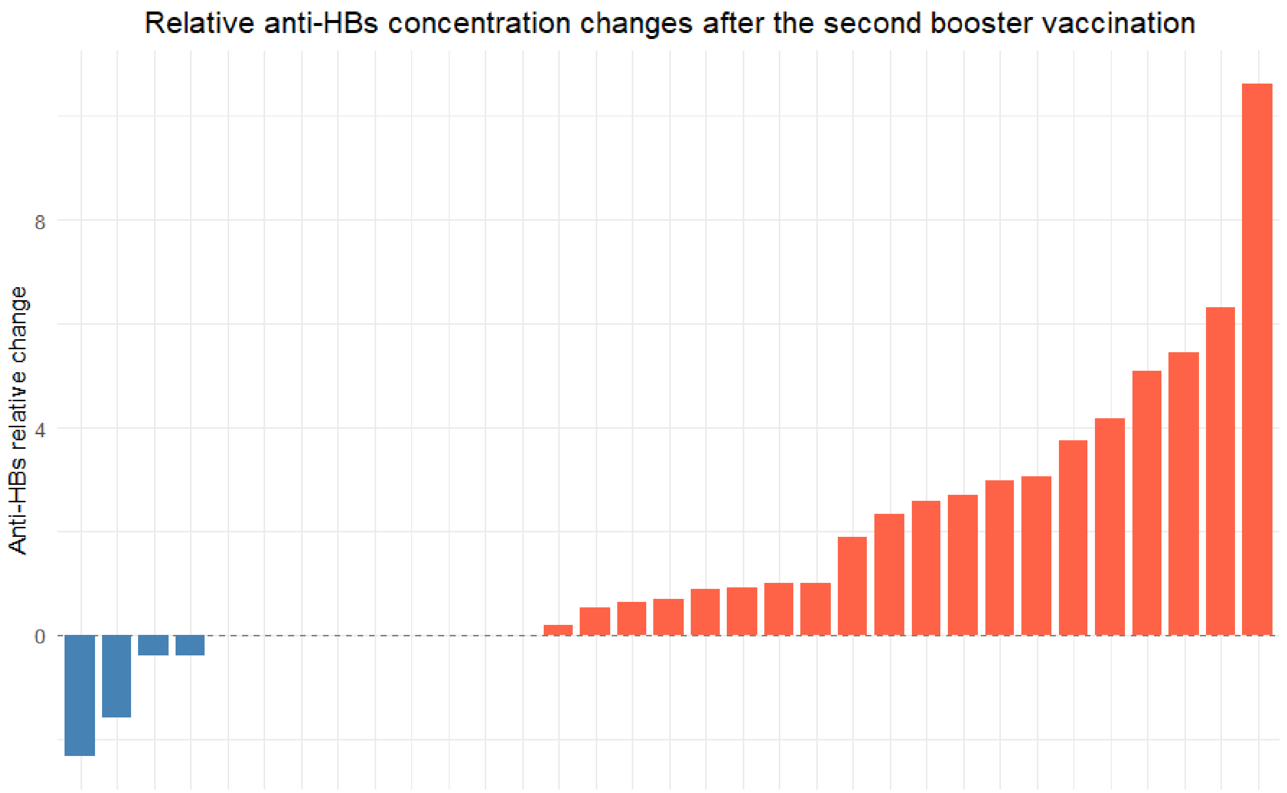

Figure S2. Relative anti-HBs concentration changes after the second booster vaccination. Plot showing the log_2 transformed relative changes in anti-HBs concentrations after the second booster vaccine compared to before the second booster vaccine. Each bar on the x-axis represents one SOT recipient. The y-axis represents log_2 transformed relative changes. Positive changes are shown in red, negative changes are shown in blue.
